# Supplementary material for: Campycins are novel broad-spectrum antibacterials killing Campylobacter jejuni
Source: Appl Microbiol Biotechnol. 2024 Oct 9;108(1):484. doi: 10.1007/s00253-024-13317-w (PMC11464564; doi:10.1007/s00253-024-13317-w)
Supplement: Supplementary file 1 — Supplementary file1 (PDF 1728 KB) [file 253_2024_13317_MOESM1_ESM.pdf]

Applied Microbiology and Biotechnology

Campycins are novel broad-spectrum antibacterials killing *Campylobacter jejuni*

Athina Zampara[1], Yilmaz Emre Gencay[1*], Lone Brøndsted[1] and Martine Camilla Holst Sørensen[1#]

[1]Department of Veterinary and Animal Sciences, University of Copenhagen, Frederiksberg, Denmark

#Correspondence: Martine Camilla Holst Sørensen, mcp@sund.ku.dk

*Present address: SNIPR Biome, Lersø Parkallé 44, København, 2100, Denmark

Supplementary Information – Supplementary figures and tables

Fig. S1 Conservation of *C. jejuni* CJIE1 H-fibers in CAMSA strains.
Fig. S2 Conservation of putative H-fiber chaperones in CJIE1 prophages in *C. jejuni* RM1221 and CAMSA2147.
Fig. S3 Structural prediction of the CAMSA2147 (green) and RM1221 (blue) H-fiber homotrimers (truncated to the stem and distal knob structure) and their chaperones (full length, trimeric form) complexes.
Fig. S4 Full SDS-PAGE images presented in Fig. 3 of pull-down assays with purified H-fibers and cell lysates from *C. jejuni* CAMSA2147 and RM1221.

Table S1 Bacterial strains.
Table S2 Plasmids.
Table S3 Primers.
Table S4 Colab AlphaFold2 confidence scores of generated models.
Table S5 Protein identification of pull-down assay SDS fragments by MS analyses.

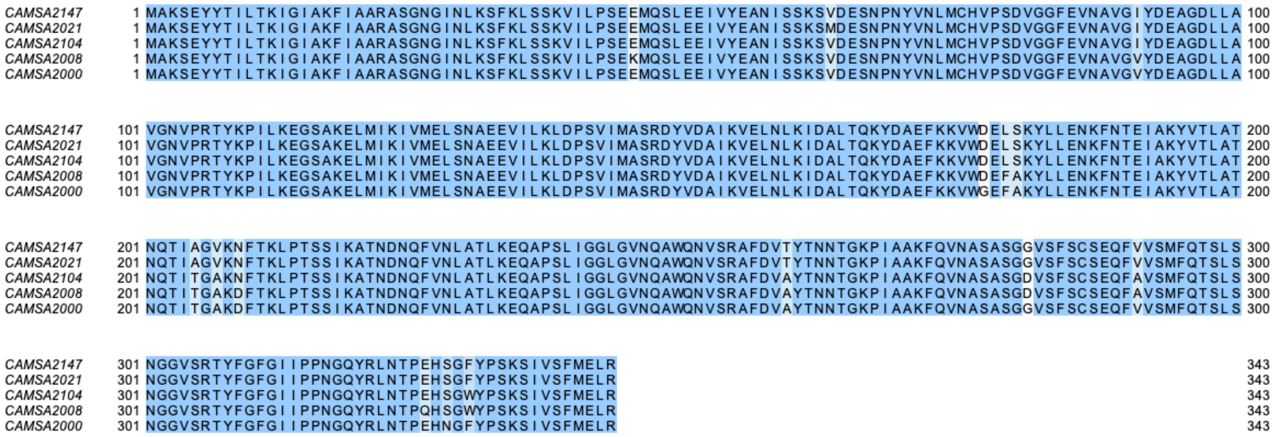

Fig. S1 Conservation of *C. jejuni* CJIE1 H-fibers in CAMSA strains. Alignment of CJIE1 prophage H-fibers present in our CAMSA strain collection. H-fiber was present in CAMSA2147 (SAMN08987259), CAMSA2021 (SAMN08987255), CAMSA2104 (SAMN08987261), CAMSA2008 (SAMN08987264) and CAMSA2000 (SAMN08987263). Amino acids are colored based on the level of conservation, with a transition from yellow to blue to represent increasing conservation. Protein sequences were aligned using CLC Main Workbench 22 (QIAGEN).

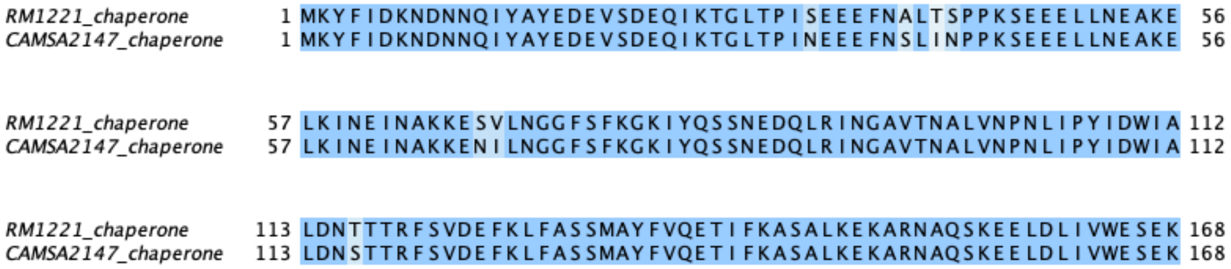

Fig. S2 Conservation of putative H-fiber chaperones in CJIE1 prophages in *C. jejuni* RM1221 and CAMSA2147. Amino acids are colored based on the level of conservation, with a transition from yellow to blue to represent increasing conservation. Protein sequences were aligned using CLC Main Workbench 22 (QIAGEN).

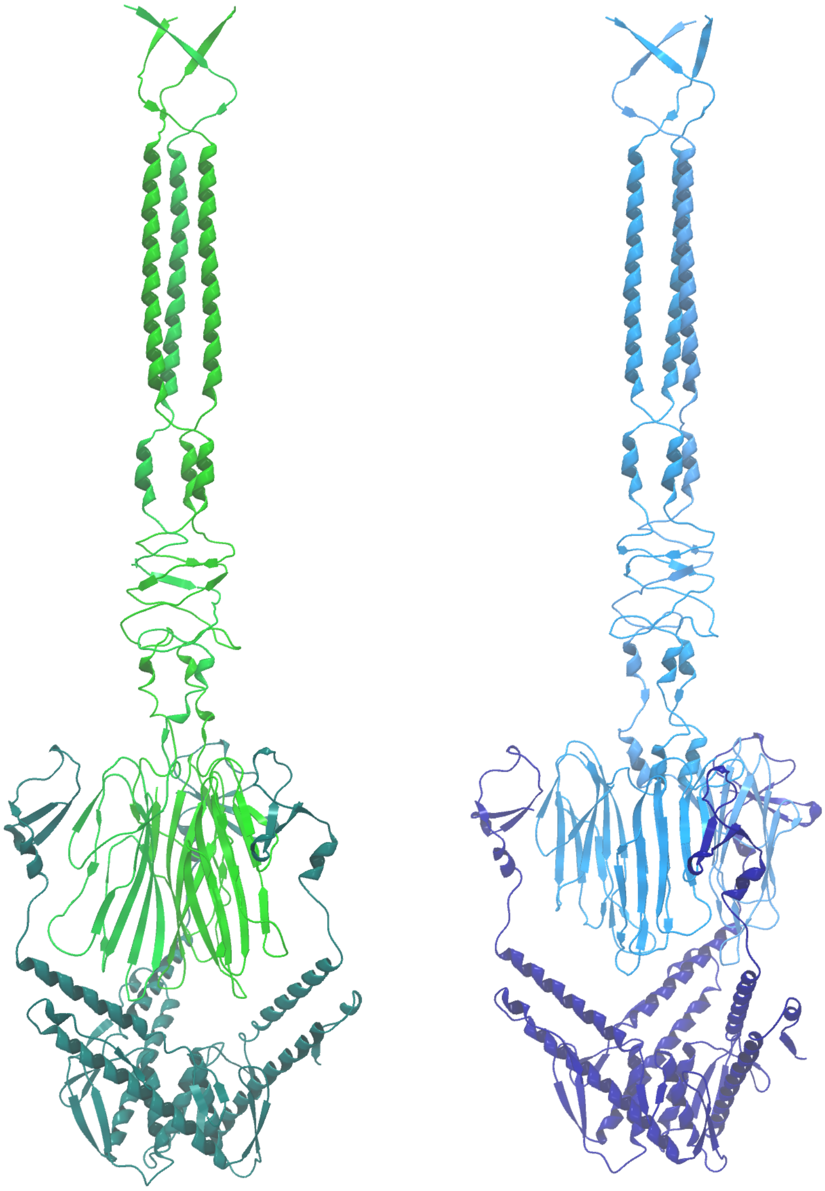

Fig. S3 Structural prediction of the CAMSA2147 (green) and RM1221 (blue) H-fiber homotrimers (truncated to the stem and distal knob structure) and their chaperones (full length, trimeric form) complexes. Model confidence scores are provided in Table S4. Proteins were visualized using CLC Main Workbench 22 (QIAGEN). Light green: CAMSA2147 truncated H-fiber, dark green: CAMSA2147 H-fiber chaperone, light blue: RM1221 truncated H-fiber, dark blue: RM1221 H-fiber chaperone.

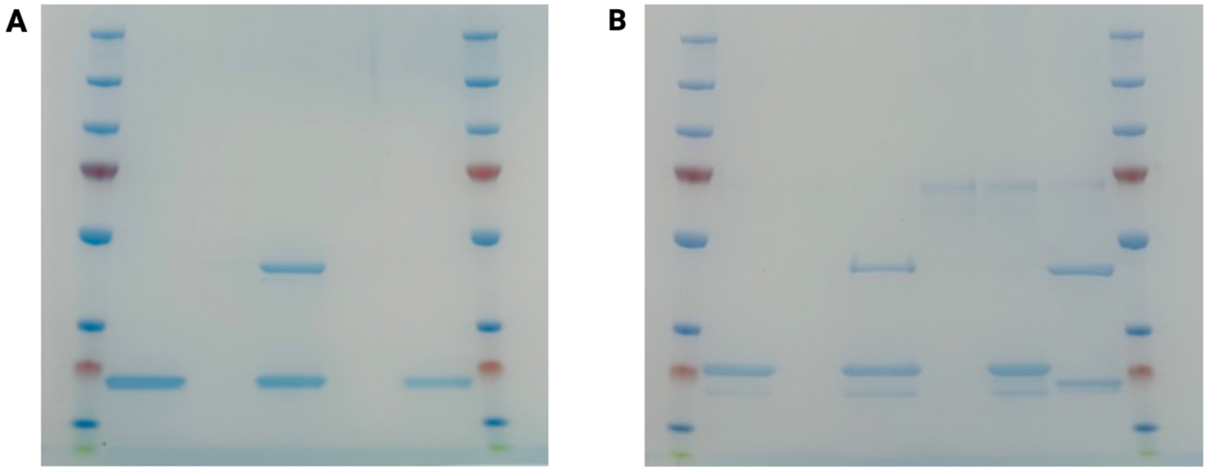

Fig. S4 Full SDS-PAGE images presented in Figure 3 of pull-down assays with purified H-fibers and cell lysates from *C. jejuni* CAMSA2147 and RM1221. (**A**) Pull-down assay with purified CAMSA2147 H-fiber. Lane 1: marker, lane 2: CAMSA2147 H-fiber co-expressed with the chaperone, lane 3: CAMSA2147 cell lysate, lane 4: CAMSA2147 cell lysate incubated with CAMSA2147 H-fiber co-expressed with the chaperone, lane 5: RM1221 cell lysate, lane 6: RM1221 cell lysate incubated with CAMSA2147 H-fiber co-expressed with the chaperone, lane 7: marker. (**B**) Pull-down assay with purified RM1221 H-fiber. Lane 1: marker, lane 2: RM1221 H-fiber co-expressed with the chaperone, lane 3: RM1221 cell lysate, lane 4: RM1221 cell lysate incubated with RM1221 H-fiber co-expressed with the chaperone, lane 5: CAMSA2147 cell lysates, lane 6: CAMSA2147 cell lysates incubated with RM1221 H-fiber co-expressed with the chaperone, lane 7: unrelated sample, lane 8: marker.

Table S1 Bacterial strains

***P. aeruginosa* strains**	**Description**			**Reference**
PAO1	Clinical wound isolate; R2-pyocin producer			Williams et al. 2008
PAO1 Δ*prf15*	*P. aeruginosa* PAO1 lacking the R2-pyocin tail fiber due to an in-frame deletion of codons 11 to 301 of *prf15*			Williams et al. 2008
PEG02	PAO1 Δ*prf15*/pM63, campycin 1 + chaperone co-expression strain, Gm^r (15 μg/ml)			Zampara et al. 2021
PEG16	PAO1 Δ*prf15*/pM96, campycin 1 expression strain, Gm^r (15 μg/ml)			Zampara et al. 2021
PEG61	PAO1 Δ*prf15*/pM179, campycin 2 expression strain, Gm^r (15 μg/ml)			This study
PEG63	PAO1 Δ*prf15*/pM182, campycin 2 + chaperone co-expression strain, Gm^r (15 μg/ml)			This study
***E. coli* strains**				
BL21-CodonPlus (DE3)-RIL	Contains a ColE1-compatible, pACYC-based plasmid containing extra copies of the argU, ileY, and leuW tRNA genes and was used as a protein expression strain, Cam^r (50 μg/ml)			Agilent Technologies
M191	BL21-CodonPlus (DE3)-RIL/CRYS1, C-terminal of CAMSA2147 *H-fiber* expression strain, Kan^r (100 μg/ml), Cam^r (50 μg/ml)			This study
M195	BL21-CodonPlus (DE3)-RIL/CRYS3, C-terminal of CAMSA2147 *H-fiber+chaperone* co-expression strain, Kan^r (100 μg/ml), Cam^r (50 μg/ml)			Zampara et al. 2021
M212	BL21-CodonPlus (DE3)-RIL/CRYS4, C-terminal of RM1221 *H-fiber+chaperone* co-expression strain, Kan^r (100 μg/ml), Cam^r (50 μg/ml)			This study
M213	BL21-CodonPlus (DE3)-RIL/CRYS5, C-terminal of RM1221 *H-fiber* expression strain, Kan^r (100 μg/ml), Cam^r (50 μg/ml)			This study
***C. jejuni* strains**	**Source**	**MLST-ST**	**ENA accession number**	**Reference**
CAMSA2052	Broiler, DK	2314	Not published	Sørensen et al. 2021
CAMSA2080	Broiler, DK	4751	Not published	Sørensen et al. 2021
CAMSA2002	Broiler, DK	21	SAMN08987254	Sørensen et al. 2021
CAMSA2021	Broiler, DK	21	SAMN08987255	Sørensen et al. 2021
CAMSA2038	Broiler, DK	21	SAMN08987256	Sørensen et al. 2021
CAMSA2086	Broiler, DK	21	SAMN08987257	Sørensen et al. 2021
CAMSA2112	Broiler, DK	21	SAMN08987258	Sørensen et al. 2021
CAMSA2147	Broiler, DK	21	SAMN08987259	Sørensen et al. 2021
CAMSA2175	Broiler, DK	21	SAMN08987260	Sørensen et al. 2021
CAMSA2104	Broiler, DK	50	SAMN08987261	Sørensen et al. 2021
CAMSA2068	Broiler, DK	22	SAMN08987262	Sørensen et al. 2021
CAMSA2093	Broiler, DK	257	Not published	Sørensen et al. 2021
CAMSA2076	Broiler, DK	400	Not published	Sørensen et al. 2021
CAMSA2003	Broiler, DK	42	Not published	Sørensen et al. 2021
CAMSA2000	Broiler, DK	45	SAMN08987263	Sørensen et al. 2021
CAMSA2008	Broiler, DK	45	SAMN08987264	Sørensen et al. 2021
CAMSA2078	Broiler, DK	45	SAMN08987265	Sørensen et al. 2021
CAMSA2138	Broiler, DK	45	SAMN08987267	Sørensen et al. 2021

CAMSA2098	Broiler, DK	583	Not published	Sørensen et al. 2021
CAMSA2118	Broiler, DK	1326	Not published	Sørensen et al. 2021
CAMSA2020	Broiler, DK	48	Not published	Sørensen et al. 2021
CAMSA2109	Broiler, DK	677	SAMN08987268	Sørensen et al. 2021
CAMSA2136	Broiler, DK	692	Not published	Sørensen et al. 2021
CAMSA2043	Broiler, DK	441	Not published	Sørensen et al. 2021
CAMSA2053	Broiler, DK	2882	Not published	Sørensen et al. 2021
CAMSA2054	Broiler, DK	354	Not published	Sørensen et al. 2021
CAMSA2096	Broiler, DK	45	Not published	Sørensen et al. 2021
CAMSA2050	Broiler, DK	1911	SAMN08987269	Sørensen et al. 2021
CAMSA2025	Broiler, DK	4748	Not published	Sørensen et al. 2021
NCTC11168	Human	43	SAMN00109763	Parkhill et al. 2000
1447 (MP9)	Chicken	475	SAMN17799213	Hansen et al. 2007
NCTC12662	Chicken	5843	SAMN06449041	National collection of type cultures
81116	Human	267	SAMN00178348	Palmer et al. 1983
81-176	Human	604	SAMN14933897	Black et al. 1988
NCTC12658	Not known	50	SAMN20837287	National collection of type cultures
RM1221	Chicken	354	SAMN14933898	Fouts et al. 2005

Table S2 Plasmids.

Plasmid	Description	Used for	Reference
**pM50**	pUCPtac::N-terminus of *prf15* of R2-pyocin lacking the binding domain, Gm^r (15 µg/ml)	Engineering R2-pyocins	Zampara et al. 2021
**pM179**	pM50::C-terminus of *H-fiber gene* of RM1221 CJIE1, Gm^r (15 µg/ml)	Campycin 2 expression	This study
**pM182**	pM50::C-terminus of *H-fiber gene* + *chaperone gene* of RM1221 CJIE1, Gm^r (15 µg/ml)	Campycin 2 co-expression with chaperone	This study
**pET28a+**	N-His, N-Thrombin, C-His, Kan^r (100 µg/ml)	Expression vector	Novagen
**pCRYS1**	pET-28 a (+)::C-terminus of *H-fiber gene* of CAMSA2147 CJIE1, Kan^r (100 µg/ml)	CAMSA2147 H-fiber expression	This study
**pCRYS3**	pET-28 a (+)::C-terminus of *H-fiber gene* +*chaperone gene* of CAMSA2147 CJIE1, Kan^r (100 µg/ml)	CAMSA2147 H-fiber with downstream chaperone expression	Zampara et al. 2021
**pCRYS4**	pET-28 a (+)::C-terminus of *H-fiber gene* +*chaperone gene* of RM1221 CJIE1, Kan^r (100 µg/ml)	RM1221 H-fiber with downstream chaperone expression	This study
**pCRYS5**	pET-28 a (+)::C-terminus of *H-fiber gene* of RM1221 CJIE1, Kan^r (100 µg/ml)	RM1221 H-fiber expression	This study

Table S3 Primers.

**Primers**	**Sequence**	**DNA amplified**	**Plasmid**
R2N Frw	AAACAGTATTCATGACGACCAATACTCCGAAATACGGT	Linearization of pM50 containing N-terminus of *prf15* of R2-pyocin lacking the binding domain	pM50
R2N Rvs	GGTACCGAGCTCTGTAAGCTTTTCCTTCACCCAGTCCTGAGTG		
RM1221 H-fiber Frw	GGTGAAGGAAAAGCTTGATGTTATTAAAGTGGAACTCAATCTTAAAATTGATGC	C-terminus of *H-fiber gene* of RM1221 CJIE1	pM179
RM1221 H-fiber Rvs	CGAGCTCTGTAAGCTTTATTGGACATTTAAAATTGTTGCCTCAATATTTGGC		
RM1221 H-fiber + chaperone Frw	GGTGAAGGAAAAGCTTGATGTTATTAAAGTGGAACTCAATCTTAAAATTGATGC	C-terminus of *H-fiber gene + chaperone gene* of RM1221 CJIE1	pM182
RM1221 H-fiber + chaperone Rvs	CGAGCTCTGTAAGCTTCATTTTTCACTCTCCCAAACAATTAAATCAAGTTC		
CAMSA2147 H-fiber Frw	CGCGCGGCAGCCATATGGCTATTAAAGTGGAACTCAATCTTAAAATTGATG	C-terminus of *H-fiber gene* of CAMSA2147 CJIE1	pCRYS1
CAMSA2147 H-fiber Rvs	GGTGGTGGTGCTCGATTATCTAAGCTCCATAAAAGATACAATGCTTTTGC		
CAMSA2147 H-fiber + chaperone Frw	CGCGCGGCAGCCATATGGCTATTAAAGTGGAACTCAATCTTAAAATTGATG	C-terminus of *H-fiber gene +chaperone gene* of CAMSA2147 CJIE1	pCRYS3
CAMSA2147 H-fiber + chaperone Rvs	GGTGGTGGTGCTCGATCATTTTTCACTCTCCCAAACAATTAAATCAAGTTC		
RM1221 H-fiber + chaperone Frw	CGCGCGGCAGCCATATGGATGTTATTAAAGTGGAACTCAATCTTAAAATTGATGC	C-terminus of *H-fiber gene +chaperone gene* of RM1221 CJIE1	pCRYS4
RM1221 H-fiber + chaperone Rvs	GGTGGTGGTGCTCGATCATTTTTCACTCTCCCAAACAATTAAATCAAGTTC		
RM1221 H-fiber Frw	CGCGCGGCAGCCATATGGATGTTATTAAAGTGGAACTCAATCTTAAAATTGATGC	C-terminus of *H-fiber gene* of RM1221 CJIE1	pCRYS5
RM1221 H-fiber Rvs	GGTGGTGGTGCTCGATTATTGGACATTTAAAATTGTTGCCTCAATATTTGGC		

Table S4 Colab AlphaFold2 confidence scores of generated models. pLLDT $\geq$ 90: high model confidence, pLLDT 90-70: confident model, pLLDT 70-50: low model confidence.

***C. jejuni* strain**	**Protein**	**pLLDT**	**Figure**
CAMSA2147	MOMP	88.5	4A
CAMSA2147	H-fiber (trimer)	79.2	5A/C
RM1221	H-fiber (trimer)	85.1	5A/C
CAMSA2147	H-fiber (trimer)-MOMP (monomer) complex	78.9	5B
CAMSA2147	H-fiber (trimer)-chaperone (trimer) complex	89.4	S3
RM1221	H-fiber (trimer)-chaperone (trimer) complex	88.9	S3

Table S5 Protein identification of pull-down assay SDS fragments by MS analyses. Matching peptides are shown in red.

**Protein sample**	**Protein description**	**Protein score**	**Protein mass**	**Protein coverage**	**Unique peptides**	**Protein sequence**
AZ1	CAMSA2147 MOMP	4168	45588	75.7%	42	MKLVKLSLVAALAAGAFSAANATPLEEAIKDVDVSGVLRYRYDTGNFDKNFVNNSNLNNSKQDHKY<br>RAQVNFSAAIADNFKAFVQFDYNAADGGYGANGIKNDQKGLFVRQLYLTYTNEDVATSVIAGKQQL<br>NLIWTDNAIDGLVGTGVKVVNNSIDGLTLAAFAVDSFMAAEQGADLLGHSNISTTSNQAPFKVDSVG<br>NLYGAAAVGSYDLAGGQFNPQLWLAYWDQVAFFYAVDAAYSTTIFDGINWTLEGAYLGNSLDSELDD<br>KTHANGNLFALKGSIEVNGWDASLGGLYYGDKEKASTVVIEDQGNLGSLLAGEEIFYTTGSRLNGDT<br>GRNIFGYVTGGYTFNETVRVGADFVYGGTKTEAANHLGGGKKLEAVARVDYKYSPKLNFSAFYSYV<br>NLDQGVNTNESADHSTVRLQALYKF
AZ2	CAMSA2147 H-fiber	3816	21173	81.8%	41	AIKVELNLKIDALTQKYDAEFKKVWDELSKYLLENKFNTEIAKYVTLATNQTIAGVKNFTKLPTSSIK<br>ATNDNQFVNLATLKEQAPSLIGGLGVNQAWQNVSRAFDVTYTNNTGKPIAAKFQVNASASGGVSFSC<br>SEQFVVSMFQTSLSNGGVSRTYFGFGIIPPNGQYRLNTPEHSGFYPSKSIVSFMELR
AZ2	CAMSA2147 H-fiber chaperone	3081	17273	100%	36	MKYFIDKNDNNQIYAYEDEVSDEQIKTGLTPINEEEFNSLINPPKSEEELLNEAKELKINEINAKKENILN<br>GGFSFKGKIYQSSNEDQLRINGAVTNALVNPNLIPYIDWIALDNSTTRFSVDEFKLFASSMAYFVQETIF<br>KASALKEKARNAQSKEELDLIVWESEK
AZ3	RM1221 MOMP	16025	46446	60%	21	MKLVKISLVAALAAGAFSAANATPLEEAIKDIDVSGVLRYRYESSNPWSNANFGSGISGKQDHKYRA<br>QVNFSGAISDNFKAFVQFDYNSQDGGYGTDSISNTSDTLTVRQLYLTYTNEDVATSVIAGKQQLNTI<br>WTDNGIDGLVGTGVKVVNNSIDGLTLAAFAMDSFNEASDTTVTITQDNNQKITGVQFNRGNPKGGS<br>DVSGALDWSKNIYGAAAIGSYDIAGGQFNPQLWLAYMSDNAFLYALDAAYSTTIFDGINWTIEGAYL<br>GNSVDNKLKDRLDAANGNFFALRGTVEVNGWDASLGGLYYGKKDKVTLTTIEDQGNLGSLLAGEEI<br>FYTNGSNLNGDIGRNIFGYVTAGYTFNETVRVGADFVYGGTKTNIIGQGGKKLEAVARVDYKYSPKL<br>NFSAFYSYVNVDTDPESTHHDAVRLQALYKF
AZ4	RM1221 H-fiber	1068	39933	30%	10	MAKSEYYTILTKIGIAKFIAARASGNGINLKSFKLSSKVILPSEEMQSLEEIVYEANISSKSVDESNPNYV<br>NLMCHVPSDVGGFEVNAVGIYDEAGDLLAVGNVPRTYKPILKEGSAKELMIKIVMELSNAEEVILKL<br>DPSVIMASRDYVDVIKVELNLKIDALTQKYDAEFKKVWDEFAKYLLENKFNTEIAKYVTLATNQTIT<br>GAKDFTKLPTSSIKATNDNQFVNLATLKENQLNLKDPELLALIGFNTYLDGTSLLKSHSMSNNVIYTN<br>TTGGKTPIAIKIQQIDNVVNQASISIYINNSLIKKINAIQYTNSTGKYIPLTHYEGILKPNDTYKIELFNIV<br>MDKPNIEATILNVQ
AZ5	RM1221 H-fiber chaperone	7417	19164	95%	20	MKYFIDKNDNNQIYAYEDEVSDEQIKTGLTPISEEEFNALTSPPKSEEELLNEAKELKIN<br>EINAKKESVLNGGFSFKGKIYQSSNEDQLRINGAVTNALVNPNLIPYIDWIALDNTTTRF<br>SVDEFKLFASSMAYFVQETIFKASALKEKARNAQSKEELD LIVWESEK

References

Black RE, Levine MM, Clements ML, Hughes TP, Blaser MJ (1988) Experimental *Campylobacter jejuni* infection in humans. J Infect Dis 157:472–479. doi: 10.1093/infdis/157.3.472.

Fouts DE, Mongodin EF, Mandrell RE, Miller WG, Rasko DA, Ravel J, Brinkac LM, DeBoy RT, Parker CT, Daugherty SC, Dodson RJ, Durkin AS, Madupu R, Sullivan SA, Shetty JU, Ayodeji MA, Shvartsbeyn A, Schatz MC, Badger JH, Fraser CM, Nelson KE (2005) Major structural differences and novel potential virulence mechanisms from the genomes of multiple *Campylobacter* species. PLoS Biol 3:e15. doi: 10.1371/journal.pbio.0030015.

Hansen VM, Rosenquist H, Baggesen DL, Brown S, Christensen BB (2007) Characterization of Campylobacter phages including analysis of host range by selected *Campylobacter* Penner serotypes. BMC Microbiol 7:90. doi: 10.1186/1471-2180-7-90.

Palmer SR, Gully PR, White JM, Pearson AD, Suckling WG, Jones DM, Rawes JC, Penner JL (1983) Water-borne outbreak of campylobacter gastroenteritis. Lancet i:287–290. doi: 10.1016/s0140-6736(83)91698-7.

Parkhill J, Wren BW, Mungall K, Ketley JM, Churcher C, Basham D, Chillingworth T, Davies RM, Feltwell T, Holroyd S, Jagels K, Karlyshev AV, Moule S, Pallen MJ, Penn CW, Quail MA, Rajandream MA, Rutherford KM, van Vliet AH, Whitehead S, Barrell BG (2000) The genome sequence of the food-borne pathogen *Campylobacter jejuni* reveals hypervariable sequences. Nature 403:665-668. doi: 10.1038/35001088.

Sørensen MCH, Gencay YE, Fanger F, Chichkova MAT, Mazúrová M, Klumpp J, Nielsen EM, Brøndsted L (2021) Identification of novel phage resistance mechanisms in *Campylobacter jejuni* by comparative Genomics. Front Microbiol 12:780559. doi: 10.3389/fmicb.2021.780559.

Williams SR, Gebhart D, Martin DW, Scholl D (2008) Retargeting R-type pyocins to generate novel bactericidal protein complexes. Appl Environ Microbiol 74:3868-3876. doi: 10.1128/AEM.00141-08.

Zampara A, Sørensen MCH, Gencay YE, Grimon D, Kristiansen SH, Jørgensen LS, Kristensen JR, Briers Y, Elsser-Gravesen A, Brøndsted L (2021) Developing innolysins against *Campylobacter jejuni*

using a novel prophage receptor-binding protein. Front Microbiol 12:619028. doi: 10.3389/fmicb.2021.619028.
